# Integration of an echocardiographic motion-management system into photon-based stereotactic arrhythmia radioablation planning

**DOI:** 10.1016/j.phro.2026.101096

**Published:** 2026-09-29

**Authors:** Hans Oppitz, Amelie L. Matusche, Laura Mantovani, Mathieu Kruska, Kerstin Siebenlist, Nour Alfakhouri, Adriano Garonna, Elisabetta Bonzano, Ester Orlandi, Viviana Vitolo, Adele Valentini, Nicoletta Basla, Frank A. Giordano, Oliver Blanck, Roberto Rordorf, Jens Fleckenstein, Judit Boda-Heggemann, Lena Kästner

**Affiliations:** a Department of Radiation Oncology, University Medical Center Mannheim, Medical Faculty Mannheim, Heidelberg University, Theodor-Kutzer-Ufer 1-3, 68167 Mannheim, Germany; b Department of Medical Physics, Istituto di Ricovero e Cura a Carattere Scientifico (IRCCS), Fondazione Policlinico San Matteo, Viale Camillo Golgi 19, 27100 Pavia, Italy; c Department of Cardiology, Haemostaseology and Medical Intensive Care, University Medical Center Mannheim, Medical Faculty Mannheim, Heidelberg University, European Center for AngioScience (ECAS), and German Center for Cardiovascular Research (DZHK) Partner Site Heidelberg/Mannheim, Theodor-Kutzer-Ufer 1-3, 68167 Mannheim, Germany; d EBAMed SA, Chemin des Mines 15, CH-1202 Geneva, Switzerland; e Department of Radiation Oncology, Istituto di Ricovero e Cura a Carattere Scientifico (IRCCS), Fondazione Policlinico San Matteo, Viale Camillo Golgi 19, 27100 Pavia, Italy; f Radiation Oncology Unit, Clinical Department, CNAO National Center for Oncological Hadrontherapy, Via Erminio Borloni, 1, 27100 Pavia, Italy; g Department of Diagnostic Medicine, Istituto di Ricovero e Cura a Carattere Scientifico (IRCCS), Fondazione Policlinico San Matteo, Viale Camillo Golgi 19, 27100 Pavia, Italy; h DKFZ-Hector Cancer Institute, University Medical Center Mannheim, Theodor-Kutzer-Ufer 1-3, 68167 Mannheim, Germany; i Mannheim Institute for Intelligent Systems in Medicine (MIISM), Medical Faculty Mannheim, Heidelberg University, Theodor-Kutzer-Ufer 1-3, 68167 Mannheim, Germany; j Department of Radiation Oncology, University Medical Center Schleswig-Holstein, Arnold-Heller-Straße 3, 24105 Kiel, Germany; k Arrhythmias and Electrophysiology Unit, Division of Cardiology, Fondazione Policlinico San Matteo, Viale Camillo Golgi 19, 27100 Pavia, Italy

**Keywords:** STAR (Stereotactic arrhythmia radioablation), Ultrasound-based cardiorespiratory tracking/ gating, Feasibility, Planning implementation

## Abstract

**Background and purpose:**

Cardiorespiratory ultrasound motion monitoring may enhance the precision of dose delivery for stereotactic arrhythmia radioablation (STAR) of ventricular tachycardia. This treatment planning study aimed to evaluate the feasibility of photon-based STAR planning in the presence of an ultrasound probe.

**Materials and methods:**

For sixteen targets in fourteen patients, three treatment plans were retrospectively created and compared: a reference plan without an ultrasound probe and two plans in which the ultrasound probe was spared from the primary treatment beam (apical or parasternal probe positions). Descriptive and statistical analyses were performed for target/organ dose-volume and treatment beam metrics, conformity-indices and maximum probe exposure.

**Results:**

The ultrasound probe could be spared from the primary beam in all cases with D_0.035 cm³_ < 1 Gy (median 0.2 Gy with apical and 0.4 Gy with parasternal placement) with slightly elevated organ dose-volume metrics and no significant differences in target dose-volume metrics. The conformity-index (50 % isodose line) differed significantly for apical and parasternal probe position (median reference plan 4.0 vs. 5.0 and 4.5, respectively; *p* < 0.01). The parasternal position gave slightly superior results compared to the apical position (e.g., heart minus planning target volume V_12.5 Gy_ 7.6 % [4.7–13.3] vs. 8.2 % [6.4–12.5]; left lung V_10 Gy_ 3.4 % [1.0–4.5] vs. 4.2 % [1.6–6.8]), albeit in all plans dose guidance parameters remained within clinically acceptable limits.

**Conclusions:**

The ultrasound probe could be spared from the primary beam without compromising photon-based STAR plan quality beyond clinically acceptable deviations. The results support further investigation of ultrasound-based STAR motion management.

## Highlights

• Photon-based stereotactic arrhythmia planning with an ultrasound probe is feasible.
• D_0.035 cm³_ to the probe below 1 Gy with apical/ parasternal probe placement.
• No significant differences in target dose-volume metrics with spared probe.
• All dose guidance parameters within clinically acceptable limits.
• Results support further investigation of ultrasound-based motion management.

## Graphical abstract

**Figure:**
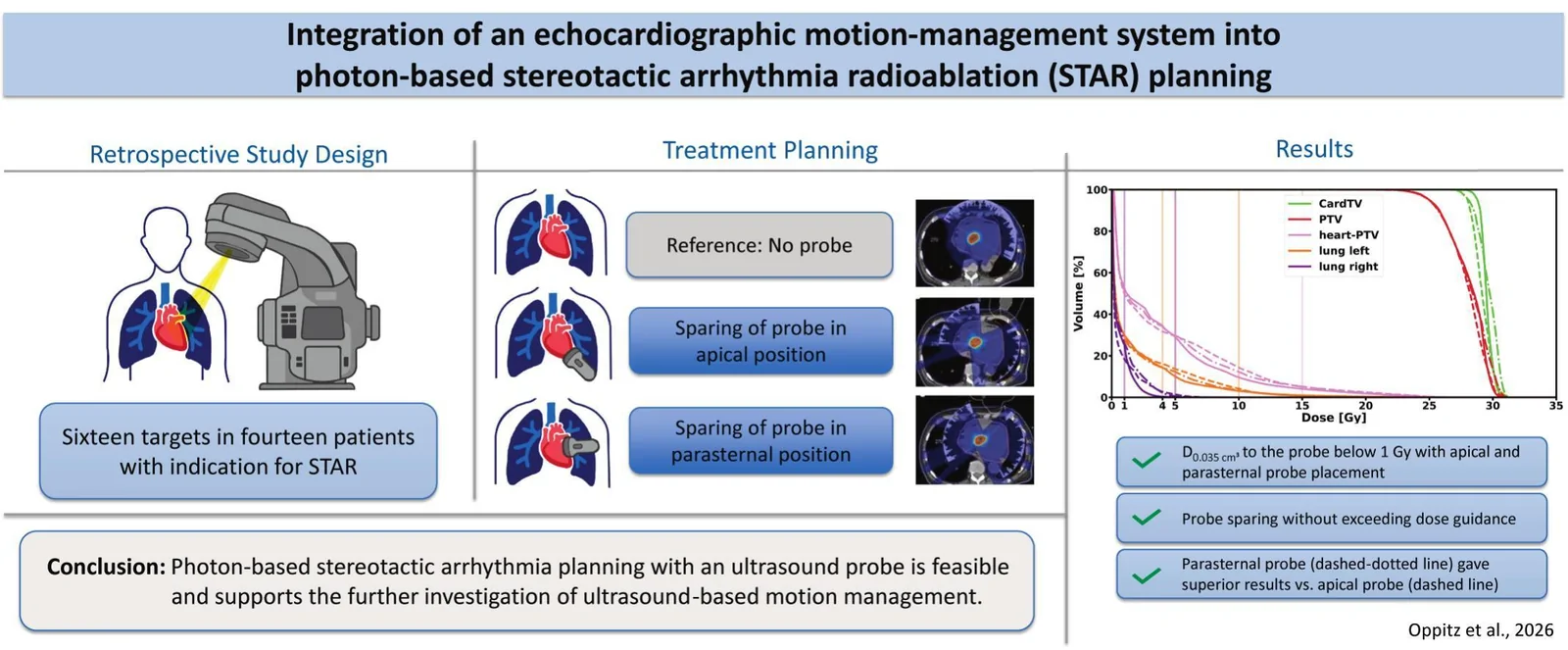

## 1 Introduction

Stereotactic Arrhythmia Radioablation (STAR) is an emerging treatment option for ventricular tachycardia (VT) [1], [2], [3]. STAR is delivered to the arrhythmogenic myocardial substrate as a single fraction of 25 Gy. Prospective trials demonstrated a substantial reduction in VT burden with a favorable safety profile [4], [5]. Ongoing clinical trials are conducted to gather further information on patterns of practice, safety, efficacy, and prognosis [6], [7], [8], [9]. For a target volume localized in the heart, respiratory and cardiac motion are relevant for precise beam delivery [10], [11], [12], [13], [14], [15]. As motion-induced uncertainties may directly compromise dose accuracy and target coverage [16], [17], active motion management (MM) is of utmost importance to ensure safe and efficient treatment [18], [19], [20]. MM approaches include respiratory gating, breath-hold, and the generation of internal target volumes [12], [13], [21], [22]. These strategies primarily address respiratory motion and fail to account for individual intrafractional cardiac/scar region motion [10], [23]. Ultrasound-based cardiorespiratory gating/tracking has been shown to be feasible in the application to abdominal and pelvic targets [24], [25], [26], [27] and is considered a promising MM strategy for STAR [28], [29].

During STAR, the ultrasound probe would be proximate to the left ventricle, therefore its presence must be considered during treatment planning. The interference between ultrasound probe and treatment beam can cause dose deviations, hence the probe should either be spared or incorporated into the dose calculation process while reducing the risk of radiation damage to the transducer [30], [31], [32], [33], [34]. Similarly, for abdominal ultrasound probes, sparing the probes from the primary treatment beam resulted in clinically unacceptable radiotherapy plans in some patients [35]. While the integration of an ultrasound probe into proton therapy STAR planning has been demonstrated [36], [37], there are currently no data on its integration into photon-based STAR with linear accelerators (linac).

This retrospective treatment planning study aimed to evaluate the feasibility of photon-based STAR planning in the presence of a hands-free ultrasound probe of an investigational echocardiographic cardiorespiratory MM system. Plans were designed to spare the ultrasound probe from the primary beam or to optimize target coverage without considering ultrasound probe location. Resulting plans were analyzed regarding target/organ of interest (OOI) dose-volume metrics, dose conformity and gradient, radiation treatment beam and plan complexity metrics, and maximum radiation exposure of technical devices (ultrasound probe, implanted cardioverter defibrillator (ICD)).

## 2 Materials and methods

Data of fourteen patients with sixteen target volumes created at two university centers within two multicenter prospective trials (the observational Italian CARA-VT dosimetry study [36], [38]; the interventional German RAVENTA study, NCT03867747 [39]) were used. All procedures were conducted in accordance with applicable laws and institutional guidelines and were approved by the local ethics board of the leading center (CARA-VT: 57629/2021; RAVENTA: 2019-406 M-§ 23b MPG und StrlSchG, including retrospective secondary analyses); written informed consent was obtained from all participants, and their privacy rights were fully respected.

### 2.1 Investigational echocardiographic cardiorespiratory motion management system

The CardioKit system (EBAMed SA, Switzerland) is an investigational echocardiographic hands-free cardiorespiratory image-guidance system for target monitoring and gated irradiation [36]. In brief, it combines automatic ultrasonographic image acquisition with an artificial intelligence (AI)-driven algorithm capable of real-time cardiac motion monitoring during radiotherapy with a mean three-dimensional variation of 1.1 mm [29]. The probe's footprint is 9 cm (diameter) and 7 cm height with attached infrared tracking tree (Fig. 1). For this planning study, apical and parasternal ultrasound positions were used, as they represent the most common ultrasound views in transthoracic echocardiography. Depending on patient-individual anatomy and ultrasound image quality, sonographic MM would be performed in only one of those two locations.

**Fig. 1:**
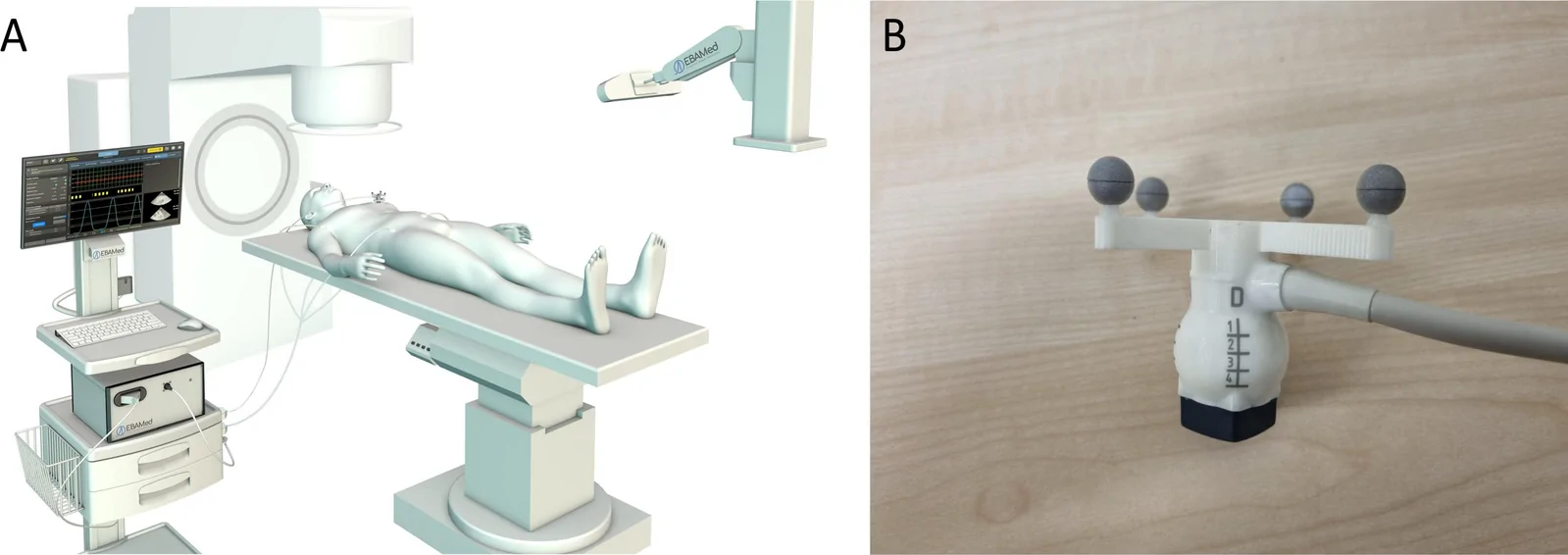
CardioKit (EBAMed SA). A: A mobile cart integrates the monitor, mouse, and Data Acquisition Module for electrocardiogram and ultrasound image capture in a linac room. B: A biplane ultrasound probe provides real-time 3D visualization of the heart and targeted ablation volume. Reflective spherical markers are tracked by a stereoscopic infrared camera in the treatment room enabling transformation of cardiac displacement information to the International Electrotechnical Commission Fixed Coordinate System for precise treatment alignment.

### 2.2 Segmentation of organs of interest, intracardiac devices and cardiac substructures

OOI (aorta, lungs, trachea, bronchi, spinal canal, skin, esophagus/stomach, superior vena cava), cardiac substructures (whole heart, left atrium, aorta, coronaries), implantable cardioverter defibrillator (ICD) aggregate, the cardiac (CardTV) and planning target volumes (PTV) had previously been contoured on each patient's planning computed tomography (CT) according to the RAVENTA study or the Italian CARA-VT protocol in Monaco V6.2.2.0 (Elekta AB, Sweden) and Raystation V·11B (Raysearch Laboratories, Sweden). To generate a homogeneous dataset, any missing structures were added and – if not already present - an isotropic 5 mm CardTV-to-PTV safety margin was created. Ultrasound probe components were segmented in Eclipse (Varian, USA) based on the skin-adhesive probe holders attached by an experienced cardiologist during planning-CT acquisition in eight of fourteen patients with nine targets (CARA-VT patient cohort within the framework of a bicentric planning study) [36]. For the remaining six patients/seven targets (RAVENTA cohort), who did not have probe holders in the planning-CT, the ultrasound probe was virtually positioned at the corresponding apical and parasternal locations on each planning-CT to simulate clinical placement for the diagnostic 2- and 4-chamber apical views and the parasternal long-axis view (Fig. S1). For each patient, the virtual positioning was compared and controlled in relation to the realistic clinical situations of the patients with skin-adhesive probe holders visible in the planning-CT. To estimate maximum dose to the probe, a water-equivalent density override was used.

### 2.3 STAR treatment planning and evaluation

For each target volume, three volumetric modulated arc therapy (VMAT) treatment plans (6 Megavoltage, Flattening-Filter-Free, 2 mm calculation grid size, Elekta Agility multileaf collimator (MLC) with 5 mm leaf thickness) were created in Monaco (V6.2.2.0, Elekta AB, Sweden). Three coplanar arcs with collimator angles of 0°, 45° and 90° for increased target coverage in inferior and superior direction were used following institutional STAR planning practice (eight patients treated so far). PTV prescription dose was 25 Gy according to the RAVENTA study protocol (PTV: D_95 %_ ≥ 25 Gy, D_2 %_ ≤ 32.5 Gy; CardTV: D_0.035 cm³_ ≥ 30 Gy; but PTV-CardTV: D_0.035 cm³_ ≤ 32 Gy instead of D_0.035 cm³_ ≤ 30 Gy [39]). A reference plan was designed, choosing the optimal VMAT arc geometry without considering the probe position. An alternative plan was optimized for each probe position (apical or parasternal long-axis echocardiographic views) to keep D_0.035 cm³_ to the probe below 1 Gy. Therefore, a planning risk volume (PRV) safety margin of 2.5 cm was used. To protect the probe from primary and exit-beam dose, gantry angles were manually excluded by avoiding PRV-PTV overlaps in beam's-eye view from anterior and, where feasible, posterior directions (Fig. 2). This often resulted in arcs being split into two halves (for fourteen and nine targets with apical and parasternal placement, respectively). If posterior PRV-PTV overlaps remained, a maximum-dose constraint for a reduced probe PRV (1.0 cm or 1.5 cm margin, water-equivalent density overwrite only during optimization) was added in critical cases (for one and five targets with apical and parasternal placement, respectively). To compensate for excluded gantry angles, the initial and final gantry angles (maximum counterclockwise and clockwise rotations, respectively) were adjusted if necessary. Thus, 48 plans were created by an experienced dosimetrist and a junior medical physicist, with consultation and evaluation by a senior medical physicist and radiation oncologist who participated in STAR planning benchmark studies [40], [41].

**Fig. 2:**
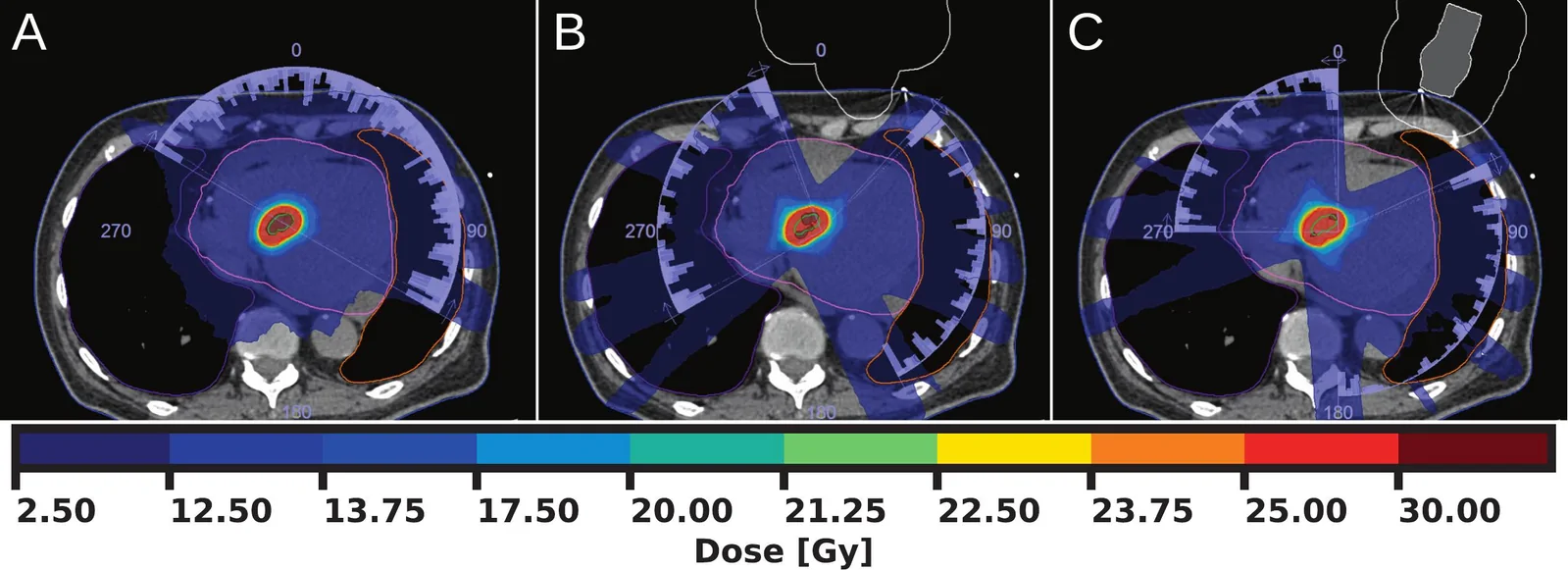
VMAT beam configurations and plan dose distribution for one PTV in the ventricular septum. A: Beam configuration without ultrasound probe. B/C: Ultrasound probe in parasternal/apical position with optimized beam geometry.

To compare plan quality, the dose-volume, dose conformity, and gradient, as well as treatment beam metrics were considered. Dose-volume histogram (DVH) metrics were analyzed for target volumes (PTV D_2 %_, PTV D_98 %_, CardTV D_0.035 cm³_, PTV-CardTV D_0.035 cm³_), OOI, cardiac substructures and ICD, focusing on potential RAVENTA major and minor protocol deviations and dose recommendations [39], [41]. Additional dose recommendations were considered for both lungs (V_20 Gy_ ≤ 10 %, V_10 Gy_ ≤ 10 %, and V_5 Gy_ ≤ 35 % [42], [43], [44]), heart-PTV (D_mean_ and V_12.5 Gy_), and stomach (D_0.035 cm³_ < 12.4 Gy [36], [45]). Finally, the median DVH showing the V_D_ sample medians (SM) in the range 0.0 Gy to 35.0 Gy was created for target, heart-PTV, and both lungs. For greater robustness, the dose-volume metric D_0.035 cm³_ was used to estimate maximum “point” dose D_max_ in this study [41], [46], [47]. Potential protocol deviations are summarized in detail in Tables S1-S3. In order to analyze the degree of dose conformity with the PTV and to quantify the steepness of the dose gradient, the ratio between the volume covered by the 95 % (CI95) and 50 % (CI50) isodose line, respectively, and the volume of the PTV was calculated [41], [48]. Beam delivery characteristics related to the spatial distribution of the arcs and delivery time (initial and final gantry angle, total arc length, sum over maximum leaf and jaw travel distances, and number of monitor units (MU)), as well as plan complexity metrics (ratio between MU and prescription dose (MU factor), plan-averaged beam area (PA) and irregularity (PI), small-aperture score (SAS(10)) and the average leaf travel (LT)) were reported [49], [50], [51], [52], [53], [54].

### 2.4 Statistical analysis

Nonzero paired differences in the metrics under consideration were evaluated statistically with a Wilcoxon-signed-rank test (5 % significance level) between the reference plans and plans with an apical or parasternal ultrasound probe position. The Python implementation scipy.stats.wilcoxon was used for the *p*-value calculation, with zero differences included in the ranking process according to the method described by Pratt et al. [55], [56], [57]. Due to the exploratory design of this treatment planning study with a small patient cohort, no correction was made for multiple testing. The *p*-values are reported descriptively and should therefore be interpreted as indicative rather than confirmative.

## 3 Results

The ultrasound probe could successfully be spared from the primary beam in all cases with D_0.035__cm³_ < 1 Gy. SM was 0.2 Gy (Interquartile Range [IQR] 0.1 Gy to 0.4 Gy, range 0.1 Gy to 0.8 Gy) with apical, and 0.4 Gy (IQR 0.2 Gy to 0.6 Gy, range 0.0 Gy to 0.9 Gy) with parasternal ultrasound probe placement.

In all plans, 25.0 Gy was prescribed to D_95 %_, except for three plans without probe, for which D_98 %_ was selected twice and D_97 %_ once to ensure that CardTV D_0.035 cm³_ exceeded 30.0 Gy. No statistically significant differences were indicated for dose-volume metrics of PTV and CardTV. The dose-gradient parameter CI50 was significantly increased for apical and parasternal probe positions, indicating a less steep dose gradient compared with the reference (Table 1). While median PTV DVH curves were almost identical, dose to the CardTV was slightly reduced with apical and slightly increased starting from 29.0 Gy with parasternal placement (Fig. 3).

**Table 1:** Sample medians [first quartile-third quartile] of dose-volume and dose conformity metrics (CI95, CI50) for all target volumes. Wilcoxon-signed-rank test was performed between the reference plan without ultrasound probe and the plans with apical and parasternal ultrasound probe positions: *: *p* < **0.05,** **: *p* < 0.01.

	Without probe	Apical probe	Parasternal probe
**PTV D**_**2 %**_**[Gy]**	30.2 [29.9–31.1]	30.3 [30.1–31.1]	30.7 [30.0–31.1]
**PTV D**_**98 %**_**[Gy]**	24.1 [23.2–24.4]	24.1 [23.7–24.3]	24.2 [23.7–24.3]
**CardTV D**_**0.035**__cm³_**[Gy]**	30.9 [30.3–31.6]	31.0 [30.7–31.6]	31.1 [30.6–31.7]
**PTV-CardTV D**_**0.035**__cm³_**[Gy]**	30.4 [30.1–30.9]	30.6 [30.5–30.9]	30.7 [30.2–31.3]
**CI95**	1.2 [1.2–1.3]	1.2 [1.2–1.3]*	1.2 [1.2–1.3]
**CI50**	4.0 [3.6–4.3]	5.0 [4.6–5.6]**	4.5 [3.9–5.5]**

**Fig. 3:**
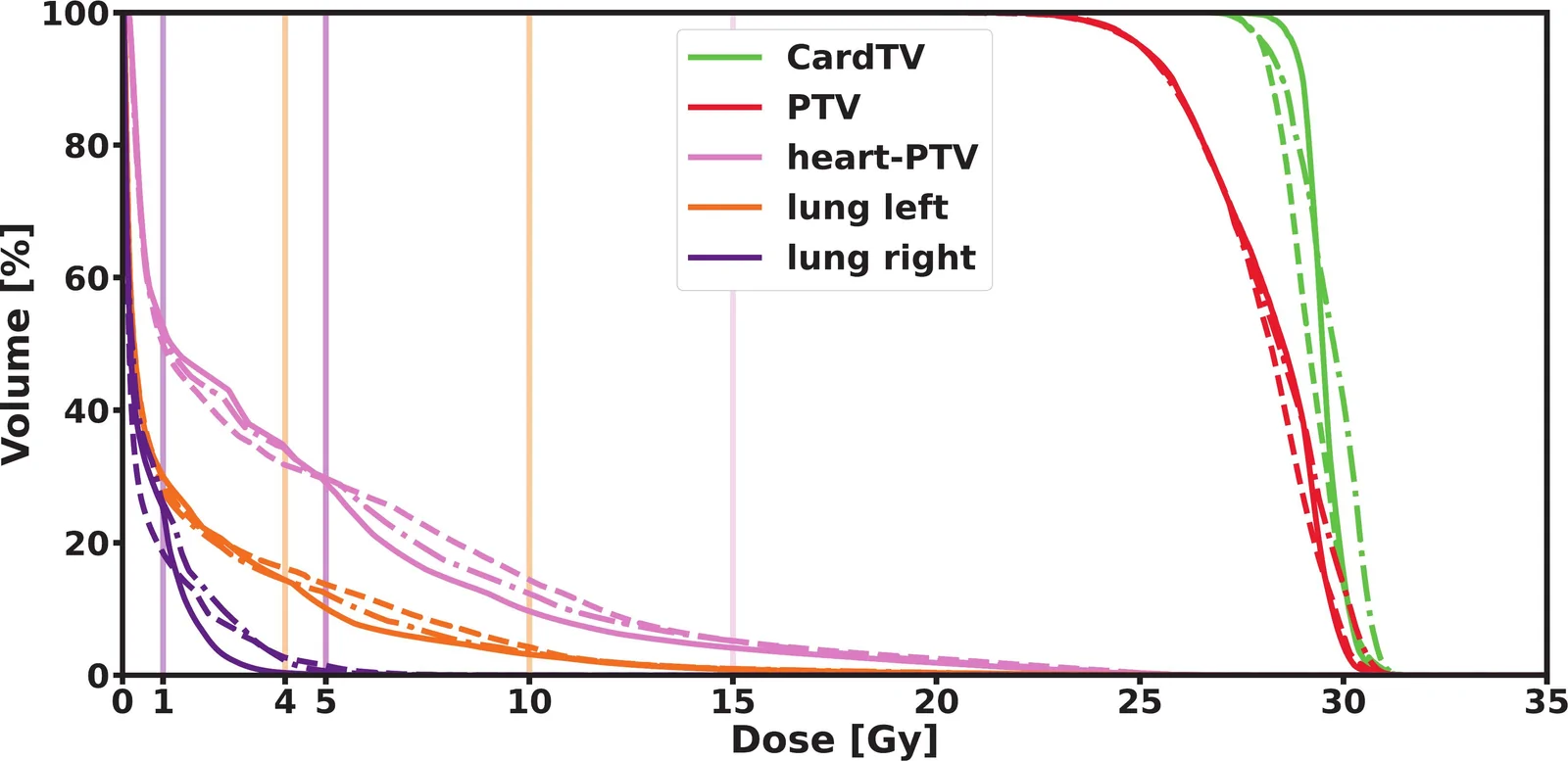
Median DVH of all target volumes (n = 16) for VMAT plans without ultrasound probe (solid line) and with apical (dashed line) and parasternal ultrasound probe position (dashed-dotted line). Vertical lines indicate regions of elevated values with ultrasound probe placement.

In general, major or minor deviations for OOI from the RAVENTA protocol only occurred for aorta and coronaries D_0.035__cm³_. Sparing of the probe did not result in additional RAVENTA protocol deviations (Tables S1, S2). A major deviation observed for aorta D_0.035 cm³_ in the reference plan was only a minor deviation with parasternal sparing. Significant differences between reference plans and plans with both apical and parasternal ultrasound placement were indicated for heart-PTV V_12.5 Gy_, skin V_10 Gy_ and D_0.035 cm³_, left lung V_10 Gy_, right lung V_5 Gy_, as well as for whole lungs D_6.5 %_, D_5 %_ and V_0 Gy_-V_7 Gy_ (Table 2). SM for left and right lung volume fractions with doses in the range 4.0 Gy to 10.0 Gy and 1.0 Gy to 5.0 Gy, respectively, as well as SM for heart-PTV volume fractions with doses between 5.0 Gy to 15.0 Gy, were slightly increased with probe sparing (Fig. 3). One apical positioned transvenous ICD (starting in the transverse plane 0.4 cm cranially from the PTV with a center-to-center distance of 7.8 cm) and one subcutaneous ICD (S-ICD and PTV with common transverse planes and a center-to-center distance of 13.2 cm) were in critical proximity to the PTV with resulting D_0.035 cm³_ of 0.2 Gy and 1.0 Gy in all plans, respectively.

**Table 2:** Sample medians [first quartile-third quartile] of dose-volume metrics for organs of interest. For statistical analysis, a Wilcoxon-signed-rank test was performed between the reference plan without ultrasound probe and the plans with apical and parasternal ultrasound probe positions: *: p < 0.05, **: p < 0.01, ***: p < 0.001, ****: p < 0.0001; ^✝^: no differences between reference plans and plans with ultrasound probe.

	**Without probe**	**Apical probe**	**Parasternal probe**
**Heart-PTV D**_**50**__**%**_**[Gy]**	1.2 [0.9–3.0]	1.0 [0.6–3.4]	1.1 [0.8–3.6]
**Heart-PTV V**_**12.5 Gy**_**[%]**	5.9 [4.4–9.0]	8.2 [6.4–12.5]**	7.6 [4.7–13.3]**
**Heart-PTV D**_**mean**_**[Gy]**	3.8 [3.2–4.8]	4.3 [3.5–4.7]	3.9 [3.2–5.0]
**Aorta D**_**0.035**__cm³_**[Gy]**	9.4 [5.7–24.7]	11.1 [5.6–24.1]	13.2 [5.7–23.1]
**Coronaries D**_**0.035**__cm³_**[Gy]**	24.0 [10.3–28.0]	25.2 [12.2–27.4]	25.4 [11.7–27.7]
**Atrium left D**_**0.035**__cm³_**[Gy]**	21.3 [5.8–27.3]	21.1 [6.6–27.4]	24.2 [6.9–27.0]*
**Superior vena cava D**_**50 %**_**[Gy]**	0.1 [0.1–0.3]	0.2 [0.1–0.3]	0.2 [0.1–0.3]**
**Esophagus V**_**9 Gy**_**[cm**^**3**^**]**	0.0 [0.0–0.2]	0.0 [0.0–0.3]	0.1 [0.0–0.3]
**Esophagus D**_**0.035**__cm³_**[Gy]**	8.0 [4.8–9.9]	8.9 [2.0–9.9]	9.2 [6.0–9.8]
**Esophagus D**_**1**__cm³_**[Gy]**	6.2 [4.0–7.8]	7.3 [1.6–8.1]	6.7 [5.1–8.1]
**Stomach V**_**9 Gy**_**[cm**^**3**^**]**	0.0 [0.0–0.0]	0.0 [0.0–0.0]^✝^	0.0 [0.0–0.0]^✝^
**Stomach D**_**0.035**__cm³_**[Gy]**	0.6 [0.5–1.3]	0.8 [0.5–1.3]*	0.7 [0.5–1.3]
**Stomach D**_**1**__cm³_**[Gy]**	0.5 [0.4–0.9]	0.7 [0.5–0.9]	0.6 [0.5–0.9]*
**Trachea V**_**9 Gy**_**[cm**^**3**^**]**	0.0 [0.0–0.0]	0.0 [0.0–0.0]^✝^	0.0 [0.0–0.0]^✝^
**Trachea D**_**0.035**__cm³_**[Gy]**	0.1 [0.1–0.2]	0.1 [0.1–0.2]	0.1 [0.1–0.2]*
**Trachea D**_**1**__cm³_**[Gy]**	0.1 [0.1–0.1]	0.1 [0.1–0.2]	0.1 [0.1–0.2]
**Bronchi V**_**9 Gy**_**[cm**^**3**^**]**	0.0 [0.0–0.0]	0.0 [0.0–0.0]	0.0 [0.0–0.0]
**Bronchi D**_**0.035**__cm³_**[Gy]**	0.3 [0.2–0.7]	0.3 [0.2–0.7]	0.3 [0.3–0.8]
**Bronchi D**_**1**__cm³_**[Gy]**	0.3 [0.2–0.4]	0.2 [0.2–0.5]	0.3 [0.2–0.4]
**Skin V**_**10 Gy**_**[cm**^**3**^**]**	0.0 [0.0–0.0]	0.6 [0.2–2.4]***	0.3 [0.0–1.1]**
**Skin V**_**14.4 Gy**_**[cm**^**3**^**]**	0.0 [0.0–0.0]	0.0 [0.0–0.0]	0.0 [0.0–0.0]^✝^
**Skin D**_**0.035**__cm³_**[Gy]**	9.0 [7.2–9.8]	12.0 [11.0–12.9]****	11.2 [9.6–11.5]***
**Lung left V**_**20 Gy**_**[%]**	0.3 [0.0–0.6]	0.3 [0.0–0.7]	0.4 [0.0–0.7]
**Lung left V**_**10 Gy**_**[%]**	3.1 [0.2–4.1]	4.2 [1.6–6.8]****	3.4 [1.0–4.5]*
**Lung left V**_**5 Gy**_**[%]**	10.1 [6.4–15.1]	13.7 [9.8–15.3]	12.3 [7.7–15.8]
**Lung right V**_**20 Gy**_**[%]**	0.0 [0.0–0.0]	0.0 [0.0–0.0]	0.0 [0.0–0.0]^✝^
**Lung right V**_**10 Gy**_**[%]**	0.0 [0.0–0.0]	0.0 [0.0–0.0]*	0.0 [0.0–0.1]
**Lung right V**_**5 Gy**_**[%]**	0.1 [0.0–0.6]	1.4 [0.9–2.9]**	0.6 [0.2–4.5]**
**Whole lungs V**_**0 Gy**_**-V**_**7 Gy**_**[cm**^**3**^**]**	4,763.0 [4,513.1–5,239.7]	4,714.7 [4,477.1–5,195.0]***	4,759.4 [4,497.6–5,181.2]**
**Whole lungs D**_**6.5 %**_**[Gy]**	4.8 [2.7–5.4]	5.2 [4.4–6.7]**	5.3 [3.6–6.1]**
**Whole lungs D**_**5 %**_**[Gy]**	5.8 [3.4–6.8]	6.6 [5.4–7.8]**	6.3 [4.1–7.3]**
**Whole lungs D**_**50 %**_**[Gy]**	0.2 [0.2–0.3]	0.2 [0.2–0.3]	0.2 [0.2–0.3]
**Spinal canal V**_**6 Gy**_**[cm**^**3**^**]**	0.0 [0.0–0.0]	0.0 [0.0–0.0]	0.0 [0.0–0.0]
**Spinal canal D**_**0.035**__cm³_**[Gy]**	2.8 [2.1–3.8]	1.1 [0.5–3.7]	2.4 [1.1–4.9]

For apical placement, initial and final gantry angles tended to be shifted counterclockwise and clockwise, respectively, compared to the reference, whereas for parasternal placement initial and final angles were shifted in both directions quite equally. While SAS(10) differed significantly from the reference for both probe positions, MU and MU factor were significantly increased for apical placement only (Table 3). Probe sparing partially led to an increase in MU for the two cases with critical ICD proximity (6,251.8 MU/15,450.1 MU for apical and 11,361.7 MU/14,217.0 MU for parasternal placement compared to 7,118.7 MU/9,671.8 MU in the reference plan).

**Table 3:** Sample medians [first quartile-third quartile] of treatment beam delivery characteristics initial/final gantry position, total arc length, sum over maximum MLC (leaves and jaws) distances, and number of MU (monitor units), as well as plan complexity metrics MU factor (MU per prescription dose), PA (plan-averaged beam area), PI (plan-averaged beam irregularity), SAS(10) (small-aperture score) and LT (average leaf travel). Wilcoxon-signed-rank test was performed between the reference plan without ultrasound probe and the plans with apical and parasternal ultrasound probe positions: *: *p* < 0.05, **: *p* < 0.01.

	**Without probe**	**Apical probe**	**Parasternal probe**
**Beam delivery characteristics**			
**Initial gantry angle [°]**	320.0 [297.5–342.5]	277.5 [260.0–286.2]**	280.0 [255.0–22.5]
**Final gantry angle [°]**	145.0 [120.0–180.0]	180.0 [160.0–180.0]*	151.0 [148.8–172.5]
**Total arc length [°]**	566.0 [502.5–600.0]	540.0 [510.0–615.0]	482.5 [450.0–564.1]
**Number of MU**	7,743.7 [7,039.7–8,601.6]	8,802.1 [7,651.2–10,804.7]**	8,514.6 [7,416.6–9,575.3]
**Max leaf travel distance [cm]**	314.9 [276.1–360.8]	302.2 [282.9–323.2]	275.9 [238.4–328.6]*
**Max Jaw travel distance [cm]**	61.0 [42.0–87.8]	55.0 [43.5–79.8]	62.2 [36.5–87.6]
**Plan complexity metrics**			
**MU factor [MU/cGy]**	3.1 [2.8–3.4]	3.5 [3.1–4.3]**	3.4 [3.0–3.8]
**PA [cm**^**2**^**]**	9.9 [8.1–11.2]	9.7 [7.7–10.5]	9.8 [7.8–10.8]
**PI**	5.1 [3.6–5.8]	4.7 [3.9–6.5]	4.7 [3.8–5.7]
**SAS(10) [%]**	15.8 [12.3–20.3]	26.2 [15.3–27.3]**	19.2 [17.6–24.5]*
**LT [cm]**	79.2 [69.4–91.2]	75.1 [63.8–89.7]	71.1 [56.5–81.0]*

## 4 Discussion

This retrospective study assessed photon-based STAR treatment planning with an ultrasound probe for cardiorespiratory MM. D_0.035 cm³_ to the ultrasound probe was less than 1 Gy. Its presence led to a less steep dose gradient and slightly elevated OOI dose-volume metrics without statistically significant differences in target dose-volume metrics. Overall, the parasternal position gave slightly superior results, albeit in all plans dose guidance parameters remained within clinically acceptable limits.

The distance between the ultrasound probe and the PTV varied among patients depending on individual anatomy and target location. Particularly when targets were located in the anterior or apical regions, beam avoidance required more pronounced modifications of arc geometry and the ultrasound probe could not always be completely shielded from exit beam dose. These findings are consistent with previous studies investigating integration of imaging devices or tracking systems into radiotherapy workflows [34], [35]. Another relevant aspect is the radiation exposure of the ultrasound probe itself. Although its electronic components could be theoretically sensitive to radiation-induced damage or performance degradation, no data on high single-fraction doses are currently available. In this study, we decided to apply the same guidance as recommended in the RAVENTA protocol for ICDs [5], [39]. For ICDs, the risk of malfunction during radiotherapy depends on factors such as photon beam energy, cumulative absorbed dose, and device type [58]. Probe sparing with a PRV margin of 2.5 cm allowed substantial dose reduction to the simulated probe structure with D_0.035 cm³_ of less than 1 Gy. This may represent a very strict precaution, but clinically acceptable plans could still be generated, indicating sufficient flexibility of VMAT-based planning.

RAVENTA dose recommendations were adhered to, except for coronaries and aorta D_0.035 cm³_, when deviations were the result of an overlap with the PTV and therefore, clinically accepted adequate target coverage was prioritized over the potential for coronary late-effects in this severely cardiac-diseased patient cohort [41]. Low to intermediate dose spread was higher with ultrasound sparing, suggesting an increased risk for major cardiac events [59]. For our current patient cohort with limited life expectancy due to severe pre-existing cardiac disease, this risk would have been clinically acceptable, given the increased safety provided by the additional image guidance during treatment. With further development of STAR and implementation for a younger, healthier patient cohort, the advantage of ultrasound image guidance should be weighed up more critically against an increased risk for cardiac late-effects. Evaluated lung dose guidance parameters were elevated with probe avoidance: For the left lung, there was a significant difference to reference plans in V_10 Gy_ and for the right lung in V_5 Gy_. However, median values were far below recommendations for both parameters. A patient-specific benefit-risk assessment of implementing an ultrasound image guidance system for better treatment accuracy and the potential increases of dose to normal tissue is essential.

Total gantry arc length and MLC travel distances indicated that treatment delivery time is almost entirely determined by the number of MU. The MU range obtained in this study is consistent with the range of 5,882 MU to 16,329 MU as it was reported in the STAR planning study from Kluge et al. [40], which is low compared to the MU needed for treatment on robotic-arm linacs (14,355 MU to 28,686 MU). Nevertheless, MU were slightly increased with probe avoidance. 6 Megavoltage Flattening-Filter-Free dose rates are nominally in the range of 1,400 MU/min [60], [61]. Therefore, MU differences in SM are expected to be less than one minute in terms of beam-on time. Two cases with critical ICD proximity to the PTV showed that MU may increase considerably more when additional geometric constraints are present.

Several limitations of this study should be acknowledged. The analysis was retrospective and partly based on simulated treatment planning data rather than actual clinical treatments with ultrasound monitoring system in place. The ultrasound probe material properties were assumed to be water-equivalent in the treatment planning system, which does not perfectly reflect the complex composition of the probe. Consequently, the calculated probe dose values should be interpreted with caution. The sample size of this study was relatively small, reflecting the still limited number of patients treated with STAR within prospective trials. Therefore, two patients contributed two targets, although shared anatomy and probe geometry may not fully support the assumption of independence. However, the analyzed dataset included patients from two independent university centers and multiple target locations within the left ventricle, thereby providing a representative range of clinical scenarios. Although parameters for the characterization of plan deliverability and complexity were reported, this cannot replace patient-specific quality assurance, which is particularly important in single fraction stereotactic treatment. Further measurements are needed to explore the extent to which the slight increase in MU factor and SAS(10) observed in this study may actually have an effect on gamma passing rates. To reduce plan complexity, it might be useful to decrease the number of arcs, enlarge the allowed minimum segment width or apply a high degree of fluence smoothing [62], [63], [64]. The impact of these parameters on dose-volume metrics, low dose spread and MU burden could be studied more systematically. Finally, the present study focused exclusively on feasibility of treatment planning and did not evaluate potential improvements in MM or treatment accuracy that could be achieved with cardiorespiratory gating. Future research should investigate the clinical integration of ultrasound-based MM systems during STAR delivery, focusing on real-time gating, workflow feasibility, and treatment-margin reduction. In this context, the different types of particle accelerators and their respective beam-on/off latencies should be considered, as beam-on latencies range from milliseconds for proton and klystron-driven photon treatment systems to approximately 1.5 s for magnetron-based travelling waveguide linacs [65], [66].

This treatment planning study demonstrated that photon-based STAR planning with avoidance of an ultrasound probe for cardiorespiratory MM is feasible without major compromises in plan quality. VMAT beam configurations can generally be adapted to spare the probe while maintaining adequate target coverage and OOI protection. These findings support further investigation of ultrasound-based real-time motion monitoring as a potential strategy to improve cardiorespiratory MM in cardiac radioablation.

## CRediT authorship contribution statement

**Hans Oppitz:** Writing – original draft, Visualization, Software, Investigation, Formal analysis, Data curation. **Amelie L. Matusche:** Writing – review & editing, Visualization, Investigation. **Laura Mantovani:** Writing – review & editing, Data curation. **Mathieu Kruska:** Writing – review & editing, Investigation. **Kerstin Siebenlist:** Writing – review & editing, Investigation. **Nour Alfakhouri:** Writing – review & editing, Software, Investigation. **Adriano Garonna:** Writing – review & editing, Supervision, Software, Resources. **Elisabetta Bonzano:** Writing – review & editing, Data curation. **Ester Orlandi:** Writing – review & editing, Data curation. **Viviana Vitolo:** Writing – review & editing, Data curation. **Adele Valentini:** Writing – review & editing, Data curation. **Nicoletta Basla:** Writing – review & editing, Data curation. **Frank A. Giordano:** Writing – review & editing, Resources. **Oliver Blanck:** Writing – review & editing, Supervision. **Roberto Rordorf:** Writing – review & editing, Data curation. **Jens Fleckenstein:** Writing – review & editing, Supervision, Investigation, Data curation, Conceptualization. **Judit Boda-Heggemann:** Writing – original draft, Project administration, Methodology, Data curation, Conceptualization. **Lena Kästner:** Writing – original draft, Visualization, Methodology, Investigation, Data curation.

## Declaration of competing interest

The authors declare the following financial interests/personal relationships which may be considered as potential competing interests: Frank Anton Giordano reports a relationship with Carl Zeiss Meditec AG that includes: funding grants, speaking and lecture fees, and travel reimbursement. Frank Anton Giordano reports a relationship with TME Pharma AG that includes: equity or stocks, funding grants, consulting or advisory, speaking and lecture fees, and travel reimbursement. Frank Anton Giordano reports a relationship with Varian Medical Systems Inc. that includes: funding grants, speaking and lecture fees, and travel reimbursement. Frank Anton Giordano reports a relationship with Siemens Healthineers AG that includes: funding grants, consulting or advisory, speaking and lecture fees, and travel reimbursement. Frank Anton Giordano reports a relationship with Guerbet SA that includes: funding grants, consulting or advisory, speaking and lecture fees, and travel reimbursement. Frank Anton Giordano reports a relationship with Cureteq AG that includes: consulting or advisory, speaking and lecture fees, and travel reimbursement. Frank Anton Giordano reports a relationship with OncomagnetTx Inc. that includes: funding grants, consulting or advisory, speaking and lecture fees, and travel reimbursement. Frank Anton Giordano reports a relationship with G-BA Innovation Fund that includes: speaking and lecture fees and travel reimbursement. Frank Anton Giordano reports a relationship with German Cancer Aid that includes: speaking and lecture fees and travel reimbursement. Frank Anton Giordano reports a relationship with CTK Poliklinik GmbH that includes: consulting or advisory, speaking and lecture fees, and travel reimbursement. Frank Anton Giordano reports a relationship with RKH Holding that includes: speaking and lecture fees and travel reimbursement. Frank Anton Giordano reports a relationship with AstraZeneca GmbH that includes: speaking and lecture fees and travel reimbursement. Frank Anton Giordano reports a relationship with FoMF GmbH that includes: speaking and lecture fees and travel reimbursement. Frank Anton Giordano reports a relationship with MEDAC GmbH that includes: speaking and lecture fees and travel reimbursement. Frank Anton Giordano reports a relationship with Elsevier GmbH that includes: speaking and lecture fees and travel reimbursement. Adriano Garonna reports a relationship with EBAmed SA that includes: employment. Jens Fleckenstein reports a relationship with EBAMed Srl that includes: consulting or advisory. Judit Boda-Heggemann reports a relationship with EBAMed Srl that includes: consulting or advisory. Judit Boda-Heggemann reports a relationship with AstraZeneca that includes: speaking and lecture fees. Lena Kaestner reports a relationship with AstraZeneca that includes: speaking and lecture fees. Frank Anton Giordano has patent licensed to Frank Anton Giordano together with Carl Zeiss Meditec AG. Frank Anton Giordano has patent licensed to Frank Anton Giordano together with TME Pharma AG. If there are other authors, they declare that they have no known competing financial interests or personal relationships that could have appeared to influence the work reported in this paper.
