# Supplementary material for: Integration of an echocardiographic motion-management system into photon-based stereotactic arrhythmia radioablation planning
Source: Phys Imaging Radiat Oncol. 2026 Sep 29;41:101096. doi: 10.1016/j.phro.2026.101096 (PMC13676723; doi:10.1016/j.phro.2026.101096)
Supplement: Supplementary file 1 [file mmc1.pdf]

Supplementary material

Integration of an echocardiographic motion-management system into photonbased stereotactic arrhythmia radioablation planning

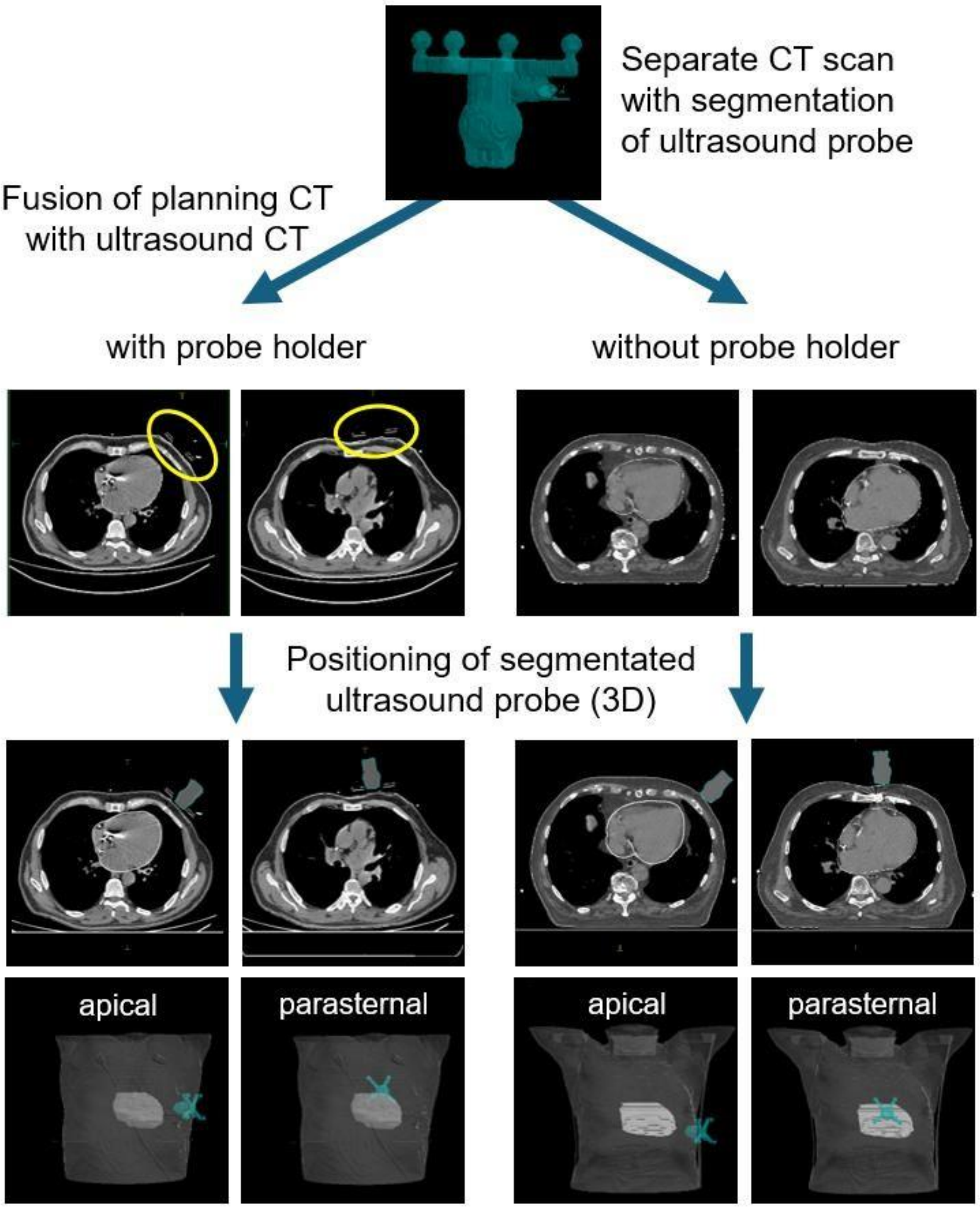

Figure S1: *Workflow of ultrasound probe positioning to pre-existing planning-CTs. A separate CT scan of the ultrasound was acquired as the ultrasound probe had been removed in all eight patients with probe holders in apical and parasternal positions to spare the probe from radiation dose. The ultrasound CT was then fused with the planning-CT in Eclipse (Varian, USA) and the segmented ultrasound probe was positioned into the probe holders by a medical physicist and verified by a senior radiation oncologist. For the remaining six patients without probe holders, the ultrasound probe was virtually positioned at the corresponding apical and parasternal locations on each planning-CT to simulate clinical placement for the diagnostic 2-and 4-chamber apical views and the parasternal long-axis view. As the aim of this study was to evaluate the general feasibility of photon-based STAR treatment in the presence of an ultrasound probe, we did not consider additional placement variability. For each patient, the virtual positioning was compared and controlled in relation to the realistic clinical situation by the vendor. To avoid possible beam attenuation of the probe itself or fiducial markers on the probe arms, a 2.5 cm PRV margin was created. Like that, the entire probe was spared from the primary beam.*

Table S1: *The respective number of treatment plans with RAVENTA major protocol deviation [1, 2].*

	Without probe	Apical probe	Parasternal probe
**Aorta $D_{0.035\ cm^3}$ > 25 Gy**	4	4	3
**Coronaries $D_{0.035\ cm^3}$ > 20 Gy**	11	11	11
**Esophagus $V_{9\ Gy}$ > 4 cm^3**	0	0	0
**Esophagus $D_{0.035\ cm^3}$ > 19 Gy**	0	0	0
**Esophagus $D_{1\ cm^3}$ > 14.5 Gy**	0	0	0
**Trachea $V_{9\ Gy}$ > 4 cm^3**	0	0	0
**Trachea $D_{0.035\ cm^3}$ > 20 Gy**	0	0	0
**Trachea $D_{1\ cm^3}$ > 15 Gy**	0	0	0
**Bronchi $V_{9\ Gy}$ > 4 cm^3**	0	0	0
**Bronchi $D_{0.035\ cm^3}$ > 20 Gy**	0	0	0
**Bronchi $D_{1\ cm^3}$ > 15 Gy**	0	0	0
**Skin $V_{14.4\ Gy}$ > 10 cm^3**	0	0	0
**Skin $D_{0.035\ cm^3}$ > 16 Gy**	0	0	0
**Whole lungs $V_{0\ Gy}$-$V_{7\ Gy}$ < 1000 cm^3**	0	0	0
**Whole lungs $D_{6.5\ \%}$ > 20 Gy**	0	0	0
**Whole lungs $D_{50\ \%}$ > 5 Gy**	0	0	0
**Spinal canal $V_{6\ Gy}$ > 1 cm^3**	0	0	0
**Spinal canal $D_{0.035\ cm^3}$ > 8 Gy**	0	0	0

Table S2: *The respective number of treatment plans with RAVENTA minor protocol deviation. Note that for bronchi and trachea $V_{9\ Gy}$ instead of $V_{10\ Gy}$ was considered, which is stricter but more in line with the respective major protocol constraints (see Table S1).*

	Without probe	Apical probe	Parasternal probe
**25 Gy ≥ Aorta $D_{0.035\ cm^3}$ > 20 Gy**	2	1	3
**20 Gy ≥ Coronaries $D_{0.035\ cm^3}$ > 14 Gy**	0	0	0
**4 cm^3 ≥ Esophagus $V_{9\ Gy}$ > 1 cm^3**	0	0	0
**19 Gy ≥ Esophagus $D_{0.035\ cm^3}$ > 14.5 Gy**	0	0	0
**4 cm^3 ≥ Trachea $V_{9\ Gy}$ > 1 cm^3**	0	0	0
**20 Gy ≥ Trachea $D_{0.035\ cm^3}$ > 15 Gy**	0	0	0
**4 cm^3 ≥ Bronchi $V_{9\ Gy}$ > 1 cm^3**	0	0	0
**20 Gy ≥ Bronchi $D_{0.035\ cm^3}$ > 15 Gy**	0	0	0
**Skin $V_{10\ Gy}$ > 10 cm^3 & $V_{14.4\ Gy}$ ≤ 10 cm^3**	0	0	0
**16 Gy ≥ Skin $D_{0.035\ cm^3}$ > 14.4 Gy**	0	0	0
**1500 cm^3 > Whole lungs $V_{0\ Gy}$-$V_{7\ Gy}$ ≥ 1000 cm^3**	0	0	0
**Whole lungs $D_{6.5\ \%}$ ≤ 20 Gy & $D_{5\ \%}$ > 20 Gy**	0	0	0
**5 Gy ≥ Whole lungs $D_{50\ \%}$ > 3.5 Gy**	0	0	0
**1 cm^3 ≥ Spinal canal $V_{6\ Gy}$ > 0.1 cm^3**	0	0	0
**8 Gy ≥ Spinal canal $D_{0.035\ cm^3}$ > 7 Gy**	0	0	0

Table S3: *The number of treatment plans that exceed considered dose recommendations [1-7].*

	**Without probe**	**Apical probe**	**Parasternal probe**
**Heart-PTV $D_{50\ \%}$ > 5.0 Gy**	1	1	1
**Atrium left $D_{0.035\ cm^3}$ > 4.4 Gy**	13	12	14
**Superior vena cava $D_{50\ \%}$ > 0.6 Gy**	0	0	1
**Stomach $D_{0.035\ cm^3}$ > 12.4 Gy**	0	0	0
**Lung left $V_{5\ Gy}$ > 35 %**	0	0	0
**Lung left $V_{10\ Gy}$ > 10 %**	0	0	0
**Lung left $V_{20\ Gy}$ > 10 %**	0	0	0
**Lung right $V_{5\ Gy}$ > 35 %**	0	0	0
**Lung right $V_{10\ Gy}$ > 10 %**	0	0	0
**Lung right $V_{20\ Gy}$ > 10 %**	0	0	0

[1] Blanck O, Buergy D, Vens M, Eidinger L, Zaman A, Krug D, et al. Radiosurgery for ventricular tachycardia: preclinical and clinical evidence and study design for a German multi-center multiplatform feasibility trial (RAVENTA). Clin Res Cardiol. 2020;109:1319–32. https://doi.org/10.1007/s00392-020-01650-9.
[2] Trojani V, Grehn M, Botti A, Balgobind B, Savini A, Boda-Heggemann J, et al. Refining Treatment Planning in STereotactic Arrhythmia Radioablation: Benchmark Results and Consensus Statement From the STOPSTORM.eu Consortium. Int J Radiat Oncol Biol Phys. 2025;121:218–29. https://doi.org/10.1016/j.ijrobp.2024.07.2331.
[3] Kaestner L, Xie J, Fanslau H, Siebenlist K, Eckl M, Oppitz H, et al. Dosimetric feasibility of stereotactic arrhythmia radioablation for ventricular tachycardia in patients with a subcutaneous implantable cardioverter defibrillator. Phys Imaging Radiat Oncol. 2025;35. https://doi.org/10.1016/j.phro.2025.100827.
[4] Sahgal A, Myrehaug SD, Siva S, Masucci GL, Maralani PJ, Brundage M, et al. Stereotactic body radiotherapy versus conventional external beam radiotherapy in patients with painful spinal metastases: an open-label, multicentre, randomised, controlled, phase 2/3 trial. Lancet Oncol. 2021;22:1023–33. https://doi.org/10.1016/s1470-2045(21)00196-0.
[5] Rossi E, Mirandola A, Basla N, Bonzano E, Ciocca M, Cisarri A, et al. Integration of ultrasound-guided motion management into proton therapy treatment plans for ventricular tachycardia noninvasive radio ablation. J Appl Clin Med Phys. 2025;26:e70213. https://doi.org/10.1002/acm2.70213.
[6] Bisello S, Cilla S, Benini A, Cardano R, Nguyen NP, Deodato F, et al. Dose-Volume Constraints fOr oRganS At risk In Radiotherapy (CORSAIR): An "All-in-One" Multicenter-Multidisciplinary Practical Summary. Curr Oncol. 2022;29:7021–50. https://doi.org/10.3390/curroncol29100552.
[7] Diez P, Hanna GG, Aitken KL, van As N, Carver A, Colaco RJ, et al. UK 2022 Consensus on Normal Tissue Dose-Volume Constraints for Oligometastatic, Primary Lung and Hepatocellular Carcinoma Stereotactic Ablative Radiotherapy. Clin Oncol (R Coll Radiol). 2022;34:288–300. https://doi.org/10.1016/j.clon.2022.02.010.
